# Oxidative Stress Induces Mitochondrial DNA Damage and Cytotoxicity through Independent Mechanisms in Human Cancer Cells

**DOI:** 10.1155/2013/825065

**Published:** 2012-12-26

**Authors:** Yue Han, Junjian Z. Chen

**Affiliations:** Division of Urology, Department of Surgery, Research Institute of McGill University Health Center, Room R1-107, 1650 Cedar Avenue, Montreal, QC, Canada H3G 1A4

## Abstract

Intrinsic oxidative stress through increased production of reactive oxygen species (ROS) is associated with carcinogenic transformation, cell toxicity, and DNA damage. Mitochondrial DNA (mtDNA) is a natural surrogate to oxidative DNA damage. MtDNA damage results in the loss of its supercoiled structure and is readily detectable using a novel, supercoiling-sensitive real-time PCR method. Our studies have demonstrated that mtDNA damage, as measured by DNA strand breaks and copy number depletion, is very sensitive to exogenous H_2_O_2_ but independent of endogenous ROS production in both prostate cancer and normal cells. In contrast, aggressive prostate cancer cells exhibit a more than 10-fold sensitivity to H_2_O_2_-induced cell toxicity than normal cells, and a cascade of secondary ROS production is a critical determinant to the differential response. We propose a new paradigm to account for different mechanisms governing cellular oxidative stress, cell toxicity, and DNA damage with important ramifications in devising new techniques and strategies in prostate cancer prevention and treatment.

## 1. Introduction

Persistent oxidative stress due to reactive oxygen species (ROS) has been associated with carcinogenesis and cancer progression [1–3], along with various aggressive cancer cell phenotypes [4]. The superoxide anion (O_2_^•−^), the primary type of ROS generated through various cellular metabolic pathways and through exposure to ionizing radiation [5], is converted into hydrogen peroxide (H_2_O_2_) and hydroxyl radical (OH^•^) via biological and antioxidant processes within the cell [6]. The hydroxyl radical, generated through the Fenton, or Haber-Weiss, reaction, is more reactive than either superoxide or hydrogen peroxide and causes direct damage to DNA and other macromolecules [7–9], resulting in DNA strand breaks and mutations.

The electron transport chain (ETC), one of the intracellular sources of ROS production, is located in the inner mitochondrial membrane and involved in the production of cellular energy through oxidative phosphorylation [10, 11]. Due to its proximity to the ETC, mitochondrial DNA (mtDNA) is sensitive to oxidative stress-related damage, which may be responsible for altered mitochondrial gene expression and somatic mutations in many human cancers [12–16]. A mitochondrial mutator phenotype has been proposed to account for the accumulation of extensive somatic mutations in clinical tumors [17]. Mitochondrial DNA is a supercoiled, closed-circular molecule with multiple copies and an average of 100 negatively superhelical turns [18]. The supercoiled structure has been identified as a functional substrate for mtDNA replication and transcription initiation in cells [19–21]. It is thus logical that disruptions to the supercoiled structure (i.e., strand breaks) would have direct effects on mitochondrial bioenergetics and mutagenesis.

In addition to DNA damage, cellular ROS can also induce cell proliferation and toxicity. The high levels of ROS generation and accumulation can lead to cell toxicity and death, making some tumor cells possible targets for ROS-induced apoptosis [22, 23], while the low levels of ROS activate signal pathways that lead to cell growth and proliferation [24]. Many human cancer cells, such as prostate, breast, colon, and malignant mesothelioma, as well as mouse colon and liver hepatoma, are shown to have increased levels of indigenous ROS and are thus under persistent oxidative stress [4, 25–27]. This may be due to upregulation of membrane-bound NAD(P)H oxidases (NOX) [28], altered energy metabolism associated with mitochondrial dysfunction [12, 29–32], and reduced antioxidant activities in superoxide dismutase and GSH pathways [6, 25]. Therefore, a bell-shaped response curve has been proposed to account for the relationship between the level of ROS and the rate of cell proliferation [25]. The increased baseline levels of ROS in tumor cells can lead to differential responses to further oxidative injury as compared to normal cells [25, 33].

In this paper, we will introduce the use of real-time PCR as a new method of assessing mitochondrial DNA damage through quantification of damaged forms (relaxed circular and linear) of supercoiled mtDNA, a concept previously introduced by our group [34]; examine some surprising interactions among cytotoxicity, ROS production, and oxidative DNA damage in prostate cancer and normal cells [33]; propose a new paradigm to explain these intriguing phenomena.

## 2. A Novel Method for Sensitive Quantification of mtDNA Damage, Repair, and Copy Number Change

The real-time PCR is a valuable tool often used to quantify starting amounts of nucleic acids in a PCR reaction without post-PCR manipulation [35, 36]. For mtDNA quantification, the relative mtDNA content is calculated as the ratio of mtDNA versus a reference nuclear gene. However, we have previously shown that different structural conformations of mtDNA (supercoiled, nicked circular, linear) have different effects on real-time PCR quantification [34]. Based on this observation, we have developed a supercoiling-sensitive quantitative PCR assay (ss-qPCR) to quantify oxidative damage in the supercoiled DNA [34, 37].

The principle of this new approach can be illustrated by model molecules. The supercoiled pBR322 plasmid DNA is previously shown to be a reliable model for mtDNA conformational studies [38]. In a comparative analysis undertaken previously [34], both supercoiled DNA molecules were digested with enzymes that altered their supercoiled DNA structure. pBR322 was treated with EcoR 1 (a single restriction site in pBR322 DNA) to generate its linear form and with N.BstNB1 (two nicking sites in pBR322 DNA) to generate a nicked (or relaxed) circular form. Total genomic DNA (containing mtDNA) from the LNCaP cells, a type of androgen responsive prostate cancer cell, was treated with EcoR 1 to linearize mtDNA. Together, two nuclear DNA, multiple mtDNA, and two plasmid DNA markers were analyzed for real-time PCR amplification using the MyiQ real-time PCR system as well as the SYBR Green I intercalation dye [34]. In this analysis, we observed that there was a 6-fold increase in the amplification of nicked circular and linear forms of plasmid DNA as compared to its untreated and supercoiled form and a 2-fold increase in the amplification of the closed-circular form of DNA when the same amount of starting template material was used (see Figure 1C in [34]). A 2-fold increase in amplification was observed in EcoR-1-treated mtDNA as compared to amplification of its untreated form in the same study. We concluded that the negatively supercoiled structure of DNA was a poor substrate for real-time PCR amplification and that a disruption of the supercoiled structure by either cutting (producing a linear molecule) or nicking (nicked circular form produced by single-strand breaks) the double-stranded molecule significantly increased the efficiency of qPCR amplification. As such, amplification efficiency can be used to determine the degree to which a supercoiled DNA sample is damaged. A heat-denaturing step at the onset of qPCR amplification can be used to introduce strand breaks into all initial mtDNA, thus enabling accurate measurement of total initial mtDNA copy number in a sample without interference from the supercoiled structure. Coupled with the quantification of DNA structural damage, the percentage of damaged mtDNA in a total sample can be calculated and the degree of oxidative stress the cell is subjected to can be inferred. A quantitative evaluation of mtDNA degradation through copy number loss can also be achieved with qPCR.

**Figure 1:**
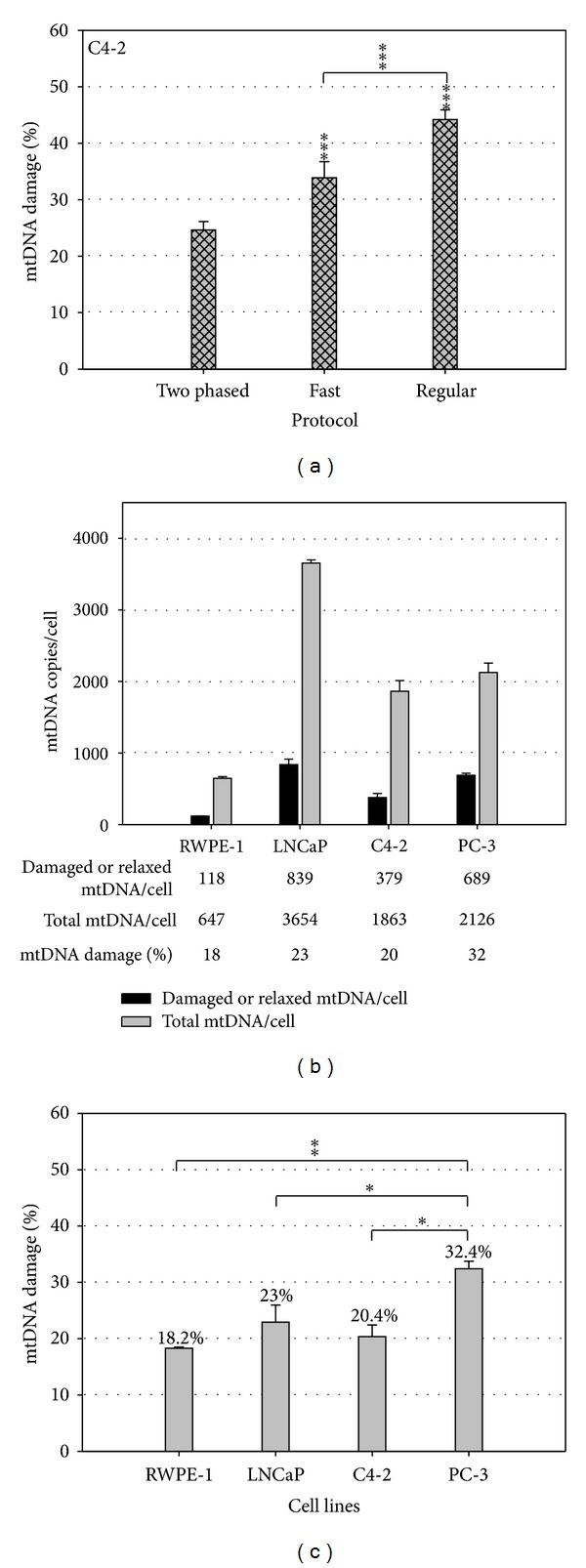
Two-phased, supercoiling-sensitive qPCR for improved mtDNA damage detection [33]. The percentage of relaxed/damaged mtDNA in C4-2 cancer cell line detected by a two-phased protocol and protocols previously reported as Fast and Regular ones (a). The absolute copy numbers of damaged and total mtDNA molecules were detected in normal RWPE-1 and three prostate cancer cell lines (LNCaP, C4-2, and PC-3) (b). The basal levels of mtDNA damage were calculated as the ratio of damaged versus total mtDNA copy numbers (c). Student's *t*-test was used for significant analysis. (*P < 0.05, **P < 0.01).

While useful, the ss-qPCR protocol used for the quantification of mtDNA damage has the potential of introducing artificial strand breaks into the sample. The initial heat-denaturing step at 95°C for three minutes, while necessary to initiate the amplification process, was observed to significantly increase qPCR amplification in mtDNA [34]. In an effort to reduce artifact, we subsequently developed a new two-step qPCR protocol with a shortened initial denaturing time at 95°C followed by a significantly lowered denaturing temperature of 80°C for the remaining cycles [33]. This 2-step procedure, by reducing both the duration and intensity of heat imposed upon the DNA substrates, led to a reduced baseline reading of mtDNA damage and increased sensitivity with regard to detecting induced mtDNA damage in different prostate cell lines. Indeed, an almost 2-fold decrease in baseline mtDNA damage levels from 44.2% to 24.6% was detected between the regular protocol and the new two-step protocol (Figure 1(a)) (Figure 1A in [33]).

The total mtDNA content per cell for different prostate cell lines and the number of damaged (or relaxed) mtDNA copies per cell can be determined using an absolute quantification approach (Figure 1(b)) (Figure 1B in [33]). Although the absolute number of mtDNA copies varies significantly, the percentage of damaged mtDNA is relatively stable across cell lines (Figure 1(c)). As such, we propose to present the level of mtDNA damage as the percentage of damaged mtDNA in the total DNA content as opposed to the absolute number of damaged molecules. Since different cell lines have different amount of mitochondria and mtDNA damage is induced within each mitochondrion, cell lines with increased levels of mitochondria may exhibit greater absolute mtDNA damage when in fact each mitochondrion has the same amount of DNA damage. Thus, the percentage of damaged mtDNA is independent from the mitochondrial content in each cell. This provides a method of evaluating the constitutively different cell lines and tissues on equal footing and gives a more accurate view into oxidative mtDNA damage between tumor and normal cells.

## 3. Differential Responses to Oxidative Injury between Prostate Cancer and Normal Cells

It is increasingly recognized that cellular ROS have a signaling role in stimulating cancer growth [2]. Therefore, intrinsic oxidative stress through enhanced levels of endogenous ROS in prostate and other cancers may be a phenotype actively selected for in cancer progression. However, which cellular processes contribute to ROS propagation in cancer and how such changes modify cancer cell responses to further oxidant injury remain to be fully elucidated. Using H_2_O_2_ as an exogenous stimulus, we previously investigated differential responses to cell toxicity, cellular ROS production, and oxidative DNA damage between prostate cancer and normal cell lines [33]. We demonstrated that aggressive prostate cancer cells exhibited a low threshold effect and increased susceptibility to extrinsic oxidative injury. Using the MTT assay, small amounts of exogenous H_2_O_2_ caused significant early redox damage and late cytotoxicity in androgen-insensitive and highly aggressive cancer cells (C4-2, PC-3) and, to a lesser extent, in androgen-sensitive cancer cells (LNCaP). However, higher doses of H_2_O_2_ were required to trigger transient cytotoxic effects in immortalized prostate epithelial cells (RWPE-1), as evidenced by a 10-fold difference in 50% growth inhibition (EC_50_) values between RWPE-1 and C4-2 cell lines [33].

Using the O_2_^•−^ specific fluorescent probe dihydroethidium (DHE), the basis of increased susceptibility to oxidative injury was shown to be associated with both a high level of endogenous O_2_^•−^ and a marked induction of secondary O_2_^•−^ production in aggressive cancer cells. Indeed, we found that the isogenic LNCaP and C4-2 cells had over 5-fold higher basal ROS levels, and PC-3, 3-fold higher levels, when compared to RWPE-1 [33]. In addition, low H_2_O_2_ doses induced persistent secondary O_2_^•−^ generation in tumor cells, yet high doses only produced transient secondary O_2_^•−^ propagation in normal cells. These findings point to a new mechanism by which exogenous H_2_O_2_ exerts its differential effects by triggering a sustained O_2_^•−^ propagation that amplifies cell toxicity in aggressive prostate cancer cells.

Cellular O_2_^•−^ is the first line of ROS production of several processes, including upregulation of membrane-bound NAD(P)H oxidases and alterations in mitochondrial respiration. We further demonstrated that the H_2_O_2_-induced O_2_^•−^ propagation specific to aggressive cancer cells is likely associated with the activation of NAD(P)H oxidases. Indeed, apocynin, a specific inhibitor of NAD(P)H oxidases, markedly reduced H_2_O_2_-induced O_2_^•−^ propagation and cytotoxicity in aggressive C4-2 cells [33]. This finding is consistent with the stimulating effect of H_2_O_2_ on NAD(P)H oxidases-mediated O_2_^•−^ production reported in human SMC, endothelial and cancer cells [39–41], and is further supported by upregulation of several isoforms of NAD(P)H oxidases in prostate cancer cell lines and tumor tissues [4, 28]. It is interesting to point out that other sources of O_2_^•−^ production downstream of NAD(P)H oxidases may be required to maintain persistent O_2_^•−^ accumulation in aggressive cancer cells, such as impaired mitochondrial respiration and oxidative DNA damage. Thus, upregulation of NAD(P)H oxidases likely confers an increased metastatic potential through enhanced levels of endogenous ROS in aggressive cancer cells, but subjects the same cells to increased susceptibility to oxidant toxicity through NAD(P)H oxidases-mediated O_2_^•−^ burst.

## 4. mtDNA Damage Is Sensitive to Exogenous H_2_O_2_ but Independent of Cellular ROS Production

The mitochondrion is both a major source of ROS production and a primary target of oxidative damage in the cell. The supercoiled mtDNA serves as a natural surrogate to oxidative DNA damage due to its close proximity to the site of ROS production [34]. The supercoiling-sensitive qPCR method provides a new opportunity to investigate whether oxidative DNA damage contributes to H_2_O_2_-induced differential cell toxicity or associates with cellular ROS production [33]. As elucidated in the MTT assay, H_2_O_2_ exposure yielded EC_50_ values of 46–112 *μ*M for prostate cancer cells (i.e., C4-2, PC-3, LNCaP) and 470 *μ*M for RWPE-1 cells after 24 h treatment, equating to a much higher resistance to oxidative stress-related cytotoxicity in normal cells when compared with cancer cell lines. However, when treated with subtoxic levels of H_2_O_2_ (30 *μ*M), RWPE-1 cells exhibited significant mtDNA damage, which increased dose-dependently with treatments of up to 240 *μ*M for 1 h. Administration of 120 *μ*M H_2_O_2_ resulted in more than 80% early structural mtDNA damage in RWPE-1 cells and induced a more than 10-fold copy number reduction in 24 h recovery, although noticeable supercoiled-structure recovery in mtDNA was also observed. Furthermore, application of 30–240 *μ*M H_2_O_2_ to C4-2 cells also resulted in prominent mtDNA damage and copy number loss. As such, exogenous H_2_O_2_ was shown to cause a very dynamic process with mtDNA damage, repair, and copy number depletion cooccurring in both prostate cancer and normal cell lines, which is in direct contrast with the differing effect it had on EC_50_ values in the different cell types. Therefore, mtDNA damage is prevalent in all cell lines but not correlated to the differential cytotoxicity between prostate cancer and normal cell lines induced by exogenous H_2_O_2_. However, it remains to be illustrated if this conclusion applies to individual cell by cell basis.

This surprising finding also suggests that acute mtDNA damage requires the presence of H_2_O_2_ and its HO^•^ derivative, but is independent of cellular O_2_^•−^ production in prostate cell lines. This observation is further supported by the lack of induced mtDNA damage in both C4-2 and RWPE-1 cell lines when treated by O_2_^•−^-producing agents, diphenyleneiodonium (DPI), and rotenone [33]. DPI is known to reduce O_2_^•−^ production by inhibiting NAD(P)H oxidases but to increase cellular O_2_^•−^ by impairing mitochondrial respiration depending on the dose and cell type used [42–44]. We demonstrated that DPI induced dose-dependent ROS production and growth inhibition in C4-2 and RWPE-1 cell lines. However, no effect was detected on either mtDNA structural damage or copy number change in both cell lines treated by DPI [33]. This result was further corroborated by the rotenone treatment that targeted specifically complex 1 of the mitochondrial ETC [33]. It is conceivable that DPI induces imbalanced O_2_^•−^ accumulation without being converted to HO^•^ through H_2_O_2_. Indeed, DPI has been shown to induce O_2_^•−^ production in many cell types [43, 44] but to suppress H_2_O_2_ production in several prostate cancer cell lines [4] and in the mitochondria of rat skeletal muscle [45]. Thus, contrary to the common assumption that cellular O_2_^•−^ is tightly balanced with H_2_O_2_ in a cell, we suggest that imbalanced accumulation of different ROS species may occur under stressed conditions, leading to very different functional consequences.

## 5. A New Paradigm of Oxidative Injury and Its Implications

**Figure 2:**
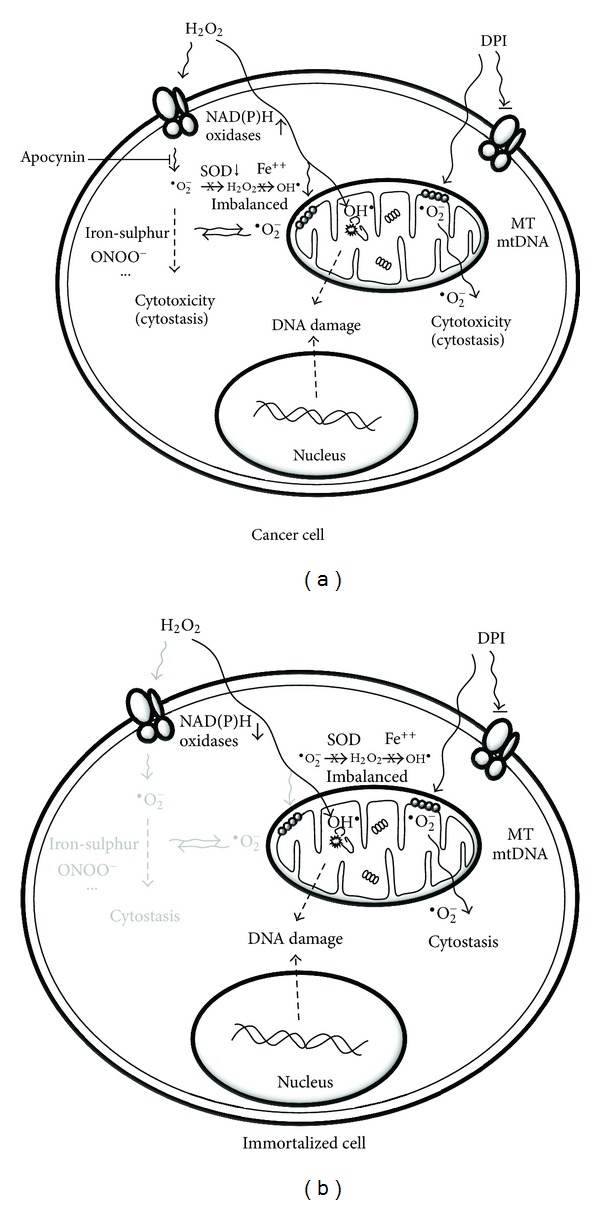
A new paradigm of oxidative injury in prostate cancer versus normal cells. Aggressive prostate cancer cells exhibit increased susceptibility to oxidant injury and DNA damage through independent mechanisms (a). See text for details. Immortalized epithelial cells are resistant to H_2_O_2_-induced cell toxicity but sensitive to oxidative DNA damage (b). The strong resistance is correlated to a lack of sustained O_2_^•−^ production through NAD(P)H oxidases, while the sensitive mtDNA damage is due to direct infiltration of H_2_O_2_ and its HO^•^ derivative.

We propose a new paradigm of oxidative injury in prostate cancer cells (Figure 2). Aggressive cancer cells exhibit intrinsic oxidative stress based on the type and source of different ROS [4, 6, 12, 25–32]. We have demonstrated that H_2_O_2_ exposure induces differential cell toxicity and sensitive oxidative DNA damage in prostate cancer cells through different mechanisms. A cascade of cellular O_2_^•−^ production is shown to be a critical determinant of selective toxicity in aggressive cancer cells [33], which is mediated by the activation of elevated NAD(P)H oxidases [28] and by crosstalk with impaired mitochondrial respiration in cancer cells [13] (Figure 2(a)). Conversely, the resistance to H_2_O_2_-induced cytotoxicity in normal cells may be attributed to a low cellular level of NAD(P)H oxidases and the normal mitochondrial function (Figure 2(b)). On the other hand, a significant level of oxidative DNA damage is induced by exogenous H_2_O_2_ in both cancer and normal cells, but is independent of cellular O_2_^•−^ steady-state levels. We propose that selective accumulation of cellular O_2_^•−^ (e.g., DPI) is cytotoxic regardless of cell types [43] but is independent to HO^•^-mediated DNA damage [33]. The O_2_^•−^ accumulation may be exacerbated by a difference in the rate of O_2_^•−^ accumulation and conversion to H_2_O_2_ and HO^•^ in stressed cancer cells. Alternatively, O_2_^•−^ may be metabolized promptly with other reactive species such as nitric oxide (NO). NO is shown to interact with O_2_^•−^ to generate peroxynitrite anions (ONOO-) and nitrogen oxides (NO_x_) [22], which could attenuate the formation of the highly reactive HO^•^ and oxidative DNA damage.

The new paradigm has important implications in designing new strategies in cancer prevention and therapy. Selective production of cellular O_2_^•−^ rather than HO^•^ is a promising strategy for preferential killing of aggressive cancer cells. This can be achieved by targeted activation of NAD(P)H oxidases in prostate cancer cells, which may be further sensitized by modulating other sources of cellular O_2_^•−^ production. In contrast, HO^•^ is a potent mutagen that may cause mtDNA damage and copy number depletion under subtoxic conditions; these active responses to DNA damage provide a new explanation to the accumulation of extensive mtDNA damage and somatic mutations in clinical tumors and aging tissues under physiological and/or pathological conditions [11, 17]. Thus, minimizing cellular HO^•^ production is better suited for cancer prevention by reducing the long-term accumulation of oxidative DNA damage and mutagenesis. Besides, the recognition of cellular partitioning and functional separation of major ROS in prostate cancer cells will likely shed new lights on the evolution of aggressive phenotype in prostate cancer. Additional investigations are desirable to test the applicability of the implications in other human cancers.

The development of a very sensitive approach to analyze structure-mediated DNA damage using real-time PCR provides a powerful, quantitative new approach to the study of mtDNA damage, repair, and copy number change in a single test [34]. This quantitative approach is in contrast to the semiquantitative analysis on mtDNA structural damage based on gel electrophoresis and southern blot [46]. Therefore, this new technical platform may find broad applications to study oxidative stress in cultured cells, clinical samples, and model animals. Indeed, mtDNA may serve as a sensitive surrogate for precise quantification of oxidative DNA damage locally in diseased tissues and systemically in circulating blood of cancer patients. We have developed a comprehensive strategy to measure multiple mtDNA end-points in circulating lymphocytes to study systemic stress in clinical investigations [47] and to study the influence of microsurgical varicocelectomy on human sperm mtDNA copy number [48]. Finally, this method also has the potential to quantify specific oxidative base lesions accumulated in supercoiled mtDNA when coupled with lesion-specific repair enzymes.

## 6. Conclusion

Cellular ROS are natural byproducts of metabolic processes, but persistent accumulation of ROS can lead to cellular oxidative injury, including DNA damage and cell toxicity. Damage to mtDNA results in the loss of its supercoiled structure, which is readily detectable with a two-step, supercoiling-sensitive qPCR assay. Our previous studies have demonstrated that mtDNA damage is very sensitive to exogenous H_2_O_2_ but independent of endogenous ROS accumulation in both prostate cancer and normal cells. In contrast, aggressive prostate cancer cells exhibit a more than 10-fold sensitivity to H_2_O_2_-induced cell toxicity than normal cells, suggesting a very different mechanism of action. We propose a new paradigm to account for different mechanisms governing oxidative stress, cell toxicity and DNA damage with important ramifications in devising new techniques and strategies in cancer prevention and treatment.
